# Comparative Analysis of Nutritional Properties, Phytochemical Profile, and Antioxidant Activities between Red and Green Water Chestnut (*Trapa natans*) Fruits

**DOI:** 10.3390/foods13121883

**Published:** 2024-06-15

**Authors:** Aniq Ur Rehman, Azmat Ullah Khan, Muhammad Sohaib, Habib Rehman

**Affiliations:** 1 Department of Food Science and Human Nutrition, University of Veterinary and Animal Science, Syed Abdul Qadir Jilani Outfall Road, Lahore 54000, Punjab, Pakistan; aniquaf987@gmail.com (A.U.R.); muhammad.sohaib@uvas.edu.pk (M.S.); 2 Department of Physiology, University of Veterinary and Animal Sciences, Syed Abdul Qadir Jilani Outfall Road, Lahore 54000, Punjab, Pakistan; habibrehman@uvas.edu.pk

**Keywords:** water chestnut, varieties, proximate compositions, comparative analysis, phytochemicals, DPPH, FRAP

## Abstract

The present study explored the nutritional composition, phytochemicals analysis, and antioxidant capacity of two indigenous varieties of red and green water chestnut (WCN) fruit grown in Pakistan. Accordingly, this study was designed to investigate the proximate composition (moisture, ash, fiber, proteins, fat, and energy), physicochemical properties (pH, °Brix, and glycemic index), minerals, and vitamins. The methanolic extracts of WCN fruits were explored for phytochemicals (total phenolic and flavonoid content), and antioxidant potential was examined in vitro by 1,1-diphenyl-2-picrylhydrazyl radical scavenging capacity (DPPH) and Ferric reducing antioxidant power (FRAP). Quantitative determination of mineral (sodium, potassium, calcium, phosphorus, iron, manganese, copper, and zinc) and vitamin (vitamin C, vitamin B6, vitamin B2, vitamin B3, vitamin A, and *β*-Carotene) composition was also assessed. Based on the findings, the proximate compositions of WCN green and red varieties varied greatly as WCN green contained significantly higher protein (1.72%), fat (0.65%), dietary fiber (2.21%), moisture (70.23%), ash (1.16%), and energy content (112.8 Kcal) than WCN red. In WCN green, the macro–micromineral concentrations were significantly higher than WCN red. Among the minerals analyzed, potassium was the most abundant mineral found in both varieties. Levels of vitamin C, B_6_, A, and *β*-Carotene were significantly higher in WCN green. In this study, methanolic extract showed higher extraction efficiency than acetone, ethanol, and distilled water. WCN green had a significantly higher quantum of total phenolic (91.13 mg GAE/g) and total flavonoid (36.6 mg QE/g) and presented significantly higher antioxidant activity than the WCN red. This study showed that, among both varieties, WCN green extract has therapeutic potential against free radical mediated health conditions and suggested the potential use of this fruit as a source of natural antioxidants in nutraceuticals.

## 1. Introduction

A diet rich in fruits and vegetables is associated with health benefits due to antioxidant and phytochemical properties. Dietary intake of fruits is crucial for human health. Their health benefits and ability to fight against diseases and infections are invaluable to humans. Fruits are a valuable dietary component as they are rich in essential nutrients such as vitamins, minerals, bioactive compounds, and phytochemicals, including antioxidants. These antioxidants are particularly beneficial as they help reduce the risk of chronic diseases [1]. Fruits are inherently high in fiber, potassium, magnesium, and vitamin C, while they are low in sodium, calories, and fat [2]. Non-conventional foods are typically consumed on special occasions and do not fall into the conventional food category, such as wild fruits [3]. The phytochemical and nutritional compositions of these conventionally available food sources have been the subject of extensive research [4]. Even though non-conventional foods are consumed widely and contribute to several health benefits. However, there is a need to evaluate the efficiency of wild and non-conventional fruit plants for comprehensive information regarding their health benefits [5]. Traditional medicinal plants contain phytochemicals, among other secondary metabolites that provide therapeutic benefits, including potent antioxidant actions [6]. In recent times, wild fruits have garnered interest as possible food supplements and cost-effective alternatives to commercially available fruits.

The water chestnut (*Trapa natans*; WCN) is a plant that floats freely and is commonly found in ponds, water-filled areas, and shallow regions in tropical and subtropical areas. According to its biogeography and ecology, it belongs to the Kingdom: Plantae, Subkingdom: *Tracheobionta*, Family: *Trapaceae*, and Genus: *Trapa*. The plant is farmed mainly for its edible fruit in the northern regions of China, Southeast Asia, and India. The most extensively grown species in Pakistan are *Trapa natans* L. and *Trapa bispinosa* Roxb [7]. WCN is a medicinal herbaceous plant renowned for its nutritional and pharmaceutical benefits [8], but its indigenous fruit varieties were previously ignored in nutritional research [9]. The WCN plant has been used in traditional medicine for centuries due its numerous health benefits [10,11]. In developing Asian countries, there has been an increasing interest in using water chestnuts to combat food insecurity [12]. WCNs have become increasingly important in ensuring food security for Southeast Asian communities in recent years. Their hardy and versatile nature allows them to thrive in flooded and dry areas, providing a reliable source of nutrition for local populations [13].

WCNs are low in calories and fat but are a good source of dietary fiber, potassium, phenols, flavonoids, and various essential minerals like iron, manganese, and potassium [14]. Therefore, it is a frequent ingredient of several famous diet plans including Mediterranean and DASH (Dietary Approaches to Stop Hypertension) diets. WCNs are popular in both fresh and processed forms worldwide. In Pakistan, fresh WCNs are mainly consumed as a fruit, while processed WCNs are used in various food products. Exploring the correlation between total phenolic content, antioxidant activity, and habitat conditions laid the groundwork for future research on safer and more affordable natural antioxidants. These antioxidants are potentially used in pharmaceutical and therapeutic preparations. The primary goal of extracting bioactive compounds from plants is to maximize both quantity and biological activity [15]. The choice of method and solvent used in the extraction dramatically impacts the results [16]. The WCN is highly valued in Indian Ayurvedic medicine for its medicinal properties. It is used to treat stomach, genitourinary, liver, kidney, and spleen issues. The plant is known for being severe, astringent, stomachic, diuretic, febrifuge, and disinfectant.

The current study was conducted to evaluate the proximate composition, physicochemical characteristics, and phytochemical potential of WCN, addressing a significant gap in current research and establishing it as a potent antioxidant ingredient in pharmaceuticals and nutraceuticals.

## 2. Materials and Methods

### 2.1. Sample Description and Reagents

Fresh WCN red and green varieties were procured from local growers representing three distinct locations of Punjab, Pakistan. Dr. A. Razaq from the Department of Biological Sciences, University of Veterinary and Animal Sciences, identified the fruit samples. Plant samples, specifically the endocarps, were meticulously washed and shade dried before being pulverized into approximately (10 × 10 mm) particles (NL7100X, NL Scientific Instruments, Klang, Malaysia). The ground samples were carefully packed into airtight bottles and stored at 4 °C for subsequent proximate analysis and estimation of phytochemical parameters. All solvents and chemicals used were of the highest analytical grade, purchased from JK Enterprises, Lahore, Pakistan. Folin–Ciocalteu’s phenol reagent (FCR), anhydrous gallic acid, methyl red, cyanidin 3-glucoside, and sodium acetate 1,1-diphenyl-2-picrylhydrazyl radical scavenging capacity (DPPH) were obtained from Merk, Darmstadt, Germany.

### 2.2. Analyses of Proximate Composition

This study followed the guidelines provided by the Association of Official Agricultural Chemists (AOAC) [17] to analyze the moisture (method # 934.06), protein (method # 945.18-B), ash (method # 942.05), fat (method # 920.85), fiber (method # 991.43), and starch (method # 996.11) content of WCNs. The AOAC method was followed with some modifications in quantifying starch.

### 2.3. Physicochemical Properties

In this study, pH measurements were conducted using a calibrated pH meter (PH-870, Tecpel, New Taipei City, Taiwan) at a temperature of 25 °C [18]. Total °Brix, a measure of sugar content, was determined using an Abbe Refractometer AR2008, (Krüss, Berlin, Germany) following the procedure outlined by [18]. Replicate samples were tested three times, and the results were reported in degrees Brix (°Brix). Additionally, the glycemic index, a measure of how quickly a food raises blood glucose levels, was determined according to the method described [19].

### 2.4. Mineral Content Analysis of Water Chestnuts

The powdered samples were digested with concentrated HNO_3_, and their mineral content was analyzed at the Sophisticated Analytical Instrumentation Centre (SAIC) of the Pakistan Council of Scientific and Industrial Research, Lahore, Pakistan. The Atomic Absorption Spectrometer (AAS-ICE 3600, Thermo Scientific, Altrincham, UK) was used for the analysis. Minerals were evaluated using the dry ash method followed by atomic absorption analysis [17].

### 2.5. Determination of Vitamin Profile

Water-soluble and fat-soluble vitamin component analysis of WCNs was performed according to the method described in [20]. For vitamin constituents, the same method was followed with some modifications. Ascorbic acid was quantified using the HPLC (Agilent technology-1260, Santa Clara, CA, USA) system. Separation was conducted using an RP-HPLC column with an isocratic mobile phase (A/B 33/67; A: 0.1 M potassium acetate, pH = 4.9, B: acetonitrile: water [50:50]) at a flow rate of 1 mL/min. Absorbance was measured at 274 nm at room temperature. The Agilent Eclipse XDB C-18 column was used for fat-soluble vitamins (5 μm, 4.6 × 250 mm). Methanol was used as a solvent. The UV detection wavelength for vitamin A and *β*-carotene was 325 nm. All vitamins were separated using isocratic elution.

### 2.6. Phytochemicals Determination of WCNs

#### 2.6.1. Extraction Process and Qualitative Phytochemical Screening of WCNs

The fruits were extracted using four different solvents, i.e., methanol, ethanol acetone, and distilled water. An amount of 50 g of air-dried fruit powder was immersed in 500 mL of different solvents and stirred for 72 h at room temperature. Afterward, the mixture was filtered (Whatman No. 1), dried in the rotary vacuum evaporator (Kalstein, Paris, France) at a temperature of 30 °C, and stored at 4 °C. The phytochemical assays were performed on all four extracts with the standard qualitative techniques for the presence of flavonoids, alkaloids, phenols terpenoids, tannin, saponin, phlobatannin, and anthraquinines [21].

#### 2.6.2. Determination of the Total Phenolic Content

The total phenolic content (TPC) of selected WCN samples was evaluated using the Folin–Ciocalteu method, as reported previously [22]. Different concentrations of the extracts (25, 50, 75, and 100 μg/mL) were meticulously prepared. The procedure for standard gallic acid was followed, and absorbance readings were performed on a spectrophotometer (V-750, Jasco, Oklahoma City, OK, USA) at 765 nm for each concentration of the extracts. The samples were analyzed in triplicate for each test, and the average absorbance value was used to plot the calibration curve to determine the level of phenolics in the extracts. The total phenolic content of the extracts was expressed as mg of gallic acid equivalents (GAE) per g of dry weight sample (mg/g). The total phenolic content in all samples was calculated using the Formula (1):

(1) $$C=c\frac{V}{m}$$

where ‘c’ shows the concentration of gallic acid determined from the calibration curve in milligrams per milliliter (mg/mL). ‘V’ represents the volume of the extract in milliliters (mL), and ‘m’ indicates the mass of the extract in grams (g).

#### 2.6.3. Determination of the Total Flavonoid Content

Total flavonoid content (TFC) was determined using the modified aluminum chloride colorimetric method as described in the literature [16,23]. A mixture containing 2 mL of the extract, 0.5 mL of 5% AlCl_3_, and 0.5 mL of 1 M potassium acetate solution was prepared following this method. The mixture was then incubated at room temperature for 15 min, with absolute ethanol used as a control. After incubation, the absorbance of all samples was measured at 415 nm using a UV–VIS spectrophotometer (V-750, Jasco, Oklahoma City, OK, USA). Quercetin was used as the reference standard for calculating the flavonoid content, which was expressed as milligrams of quercetin equivalent (QE) per gram.

### 2.7. Antioxidant Capacity Evaluation

#### 2.7.1. DPPH Radical Scavenging Activity

In vitro, free radical scavenging activity of WCN extracts was measured using the 1,1-diphenyl-2-picrylhydrazyl radical scavenging capacity (DPPH) technique described earlier [24]. Briefly, a mixture was prepared by combining 50 µL of WCN extract with 950 mL DPPH solution in methanol (0.08 mg). Subsequently, a 60 min incubation was carried out at room temperature without light. The absorbance was measured at 517 nm (spectrophotometer V-750, Jasco, Oklahoma City, OK, USA) for both the sample and the standard with a spectrophotometer. A standard was set using ascorbic acid. The results of the DPPH assay were expressed as IC_50_, representing the substrate concentration that leads to a 50% reduction in DPPH activity. This parameter helps interpret the assay results and assess the sample’s antioxidant activity. The Formula (2) used to calculate the sample’s antioxidant activity is as follows:

(2) $$DPPH\;radical\;scavenging\;ability\;\left(\%\right)=\frac{Absorbance\;of\;control-Absorbance\;of\;sample}{\begin{array}{c} Absorbance\;of\;control \end{array}}\times 100$$

#### 2.7.2. Ferric Reducing Antioxidant Power Assay

The antioxidant potential of WCN extracts was assessed using the ferric reducing antioxidant power (FRAP) assay, following the method described earlier [25,26]. This process involves the conversion of Fe^3+^ to Fe^2+^ by an antioxidant, resulting in the formation of a blue complex (Fe^2+^/TPTZ) and an increase in absorbance at 593 nm. The FRAP assay was performed by combining a buffer solution containing 300 mg of acetate (pH 3.6), 10 mM of TPTZ, 40 mM of HCl, and 20 mg of FeCl_3_ in a 10:1:1 (*v*/*v*/*v*) ratio. Sample solutions (100 mL) were mixed with the reagent (3400 mL) in each well. After a 30 min incubation period, the optical density was measured at 593 nm, and standard curves were generated using various concentrations of Trolox. The results were expressed as Trolox equivalent millimoles per gram (mmol/g).

### 2.8. Statistical Analysis

All assays were conducted in triplicate, and the results are reported as mean ± standard deviation. Statistical differences were analyzed using independent sample *t*-test with a significance level of *p* ≤ 0.05 using the SPSS version 25.0 software.

## 3. Results

### 3.1. Proximate Compositions and Physicochemical Properties

The proximate compositions of WCN varieties are presented in Table 1. The statistical analysis of the data indicated significant differences among the varieties for all proximate parameters analyzed. The results showed that moisture, ash, fiber, protein, and fat contents were significantly higher in the green fruit variety compared to the red variety. However, starch content was significantly higher in the red variety. Several important parameters related to the physicochemical properties of WCN fruits, such as pH, °Brix, energy content, and glycemic index, are summarized in Table 2. The highest energy content was recorded in the green fruit variety. However, the glycemic index of the green fruit variety was significantly lower than that of the red fruit variety.

### 3.2. Mineral Profile of Water Chestnut

Table 3 shows the mean values of macro (Na, K, Ca, and P) and micromineral contents (Fe, Mn, Cu, Zn) in green and red WCNs. The results indicate that Na, K, Ca, and P contents were significantly higher in the green fruit variety than in the red variety. Similarly, the micromineral contents were also higher in the green fruit variety compared to the red variety. However, Mn and Zn contents were significantly lower in the green WCN variety.

### 3.3. Vitamin Contents

Water-soluble vitamins analyzed, including ascorbic acid (vitamin C), pyridoxine (vitamin B6), riboflavin (vitamin B2), and niacin (vitamin B3), are expressed per 100 g of WCN in Table 4. Vitamin C and B6 were found to be ubiquitously present in both WCN varieties, while vitamin B2 and B3 were not detected in either variety. WCN green showed significantly higher content of vitamin C and B6 compared to WCN red (Table 4). Among the fat-soluble vitamins, *β*-Carotene and retinol (vitamin A) were tested. WCN green exhibited significantly higher *β*-Carotene content compared to WCN red. However, retinol (vitamin A) was not detected in either WCN variety.

### 3.4. Phytochemical Screening

Red and green WCN were extracted using four different solvents (methanol, ethanol, acetone, and aqueous) and then subjected to the qualitative phytochemical screening to identify the presence of flavonoids, alkaloids, phenols, terpenoids, tannins, saponins, phlobatannins, and anthraquinones. The phytochemical profile revealed that the various extracts of the formulation are enriched with a variety of bioactive compounds that exhibit promising pharmacological properties (Table 5 and Table 6). Flavonoids, phenols, and saponins were the most abundant classes of compounds in both varieties. Our results showed that methanolic extracts had the highest quantity of phytochemicals, followed by the ethanol, acetone, and aqueous extracts.

Results showed that different solvents resulted in significantly different extraction yields of phytonutrients. Specifically, the extracts obtained using methanol have shown the highest levels of flavonoids, phenolics, and antioxidants, resulting in the most substantial output during extraction. Overall, WCN green showed prominent greater phytoconstituent than WCN red fruit.

#### 3.4.1. Total Phenolic Content

Table 7 shows the mean values of the total phenolic content of the WCN varieties. The TPC was determined using Folin–Ciocalteu reagent and expressed as gallic acid equivalents. The TPC values varied largely among solvents, where methanolic extract achieved the highest TPC. Among the studied varieties, WCN green contained a significantly higher amount of TPC (91.1 GAE mg/g) than red (36.6 GAE mg/g). The Folin–Ciocalteu assay was conducted three times, and the calibration curve of gallic acid showed the highest absorbance at 760 nm (y = 0.0531x + 0.0003 R^2^ = 0.9951).

#### 3.4.2. Total Flavonoid Content

The TFC in both WCN green and red was measured and expressed as quercetin equivalents. The TFC was quantified using a linear equation derived from the quercetin calibration curve (y = 0.0291x − 0.0397 R^2^ = 0.9904). Different efficiencies of the extracting solvents of TFC from the WCNs were observed. The data showed that the TFC of WCN green (69.8 mg QE/g) was significantly higher than WCN red (24.1 mg QE/g) (Table 7).

### 3.5. In Vitro Antioxidant Activity

The antioxidant capacity of methanolic extracts from WCN green and red fruits was evaluated using the DPPH radical scavenging activity and FRAP methods. The scavenging activities were quantified as IC_50_ values, representing the concentration required to inhibit 50% of the DPPH. Lower IC_50_ values indicate a higher radical scavenging capacity. The results, presented in Table 7, show that the IC_50_ value of the WCN red fruit extract (50.22 ± 0.79%) was significantly lower than that of the WCN green fruit extract (62.84 ± 0.94%). The WCN green fruit extract had a higher FRAP value (42.45 ± 0.44 µM TE/g DE) than the red fruit extract (31.72 ± 0.54 µM TE/g DE) for the methanol extract, indicating that it had more potent antioxidant properties (as shown in Table 7).

## 4. Discussion

When comparing the results of proximate analysis of this present study, significant variations were observed in all tested parameters between WCN red and green varieties. The moisture values were within the range of values previously reported in India and Bangladesh. The determination of moisture is critical in the shelf life and storage of fruits [27]. The ash content indicates the presence of inorganic mineral content of fruit samples. In contrast to earlier findings, the ash contents of the investigated WCN samples were lower, possibly due to their different botanical origins [28]. Analyzing protein content is fundamental for determining the nutritional profile of any food. The protein contents of WCN varieties reported in this study were found to be lower than values reported previously [29]. A previous study reported fairly similar protein content (1.24%) of WCNs largely grown in Burma [30]. Similar variation in protein contents has been reported earlier and could be attributed to different environmental conditions and agroecological zones [31]. Fat is the most energy-dense macronutrient and an essential substance for a healthy life. A low-fat food is generally considered a healthy food and, therefore, fat content in food has a significant role in our diet [32]. The WCN contain a low amount of fat (0.54–0.65%) that makes it an ideal food in weight-reducing diets. The low fat contents in the fruit corroborates with previous findings [33] (0.52%), but are lower than fat content (2.1%) reported in another study [34]. Dietary fiber is the indigestible portion of fruit and has been linked to several health benefits such as improved metabolism, gut, and heart health. In the present study, fiber contents of WCN fruit (2.0–2.21%) were similar to the previous literature (2.13–2.27%) [35]. In contrast to our present findings, the Indian study reported the higher dietary fiber content of 4.58 g/100 g in WCNs. Furthermore, our present findings were higher than previous studies [29,33]. The WCN is a good source of starch and a viable substitute to cornstarch, exhibiting comparable properties [36,37]. The starch contents of WCN fruits (19.52–21.9%) were higher than previous reports [38,39]. In the present study, both fruit varieties were found to have a low caloric content (108.69–112.80 Kcal/100 g) in line with previous reports [40]. These results showed that WCN fruit has a low calorie count with many nutrients; thus, it could be used to promote weight loss. The glycemic index helps assess how carbohydrates affect blood sugar, crucial for diabetes management. WCN red has a higher glycemic index than green, likely due to red’s higher starch content. The data were in close agreement with a previous study [41]. Micronutrients are essential for various physiological functions and overall health. WCN fruit is rich in micronutrients such as sodium, potassium, manganese, copper, vitamin B6, and riboflavin. Current findings showed that WCN green had significantly higher mineral and vitamin contents than WCN red, which is consistent with a previous report [38]. In the present study, potassium was found to be the most abundant mineral in the WCN varieties, consistent with previous findings [30,42]. WCN has more sodium than other wild fruit varieties [43]. The findings of the current study are closely related to the outcomes of an earlier study [44] where Na concentration was documented in African WCN cultivar (3.7 mg/100 g). In WCN varieties, observed calcium contents were in accordance with the previous findings [34,40].

In the present study, phosphorus was the second most abundant mineral in WCN varieties, as reported earlier [30]. Phosphorus plays a crucial role in bone and teeth formation, as well as in regulating acid–base balance in the body [45]. Iron is an essential mineral that the human body requires for the formation of hemoglobin and red blood cells, which are responsible for transporting oxygen throughout the body as oxyhemoglobin [46]. The iron content results of the present study were lower than a previous study conducted in Bangladesh [35]. These results were higher than those previously reported in the literature [40]. Manganese is a trace mineral and essential component of several antioxidant enzymes. The amount of manganese in WCN varieties (14–15 µg/g) was in agreement with previously reported findings in the literature [35,42]. The variation in the mineral contents with previous reports can be attributed to environmental and genetic factors, including differences in soil composition between regions, which could contribute to these discrepancies. Copper and zinc are essential micronutrients and play important roles in several metabolic reactions, acting as cofactors for numerous enzymes. Copper and zinc contents reported in the present research were in line with previous data [33].

Importantly, the vitamins found in WCNs were ascorbic acid, pyridoxine, and *β*-Carotene. The content of ascorbic acid found in the present study was different from earlier reports [35,47,48]. WCN green presented higher pyridoxine (Vitamin B6) contents than the red variety. Pyridoxine plays essential roles in metabolism, neurotransmitter synthesis, immune support, and various other physiological functions [49]. WCNs provide a notable amount of pyridoxine, with 17% of the Recommended Daily Intake (RDI). *β*-Carotene is a precursor to vitamin A, which is essential for vision, immune function, and skin health [50]. The WCN is not particularly rich in *β*-Carotene (39–46 μg/100 g) compared to some other fruits and vegetables. These results were slightly lower than those reported in the literature by [35] (60–92 μg/100 g) for chestnut fruits from Bangladesh.

Phytochemicals possess remarkable antioxidant properties and are known to have several health benefits such as anti-inflammatory effects, immune system support, and potential protective effects against certain diseases. [51]. The most important step prior to quantification of phytochemicals is the extraction stage [52]. The solvents used for photochemical extractions have a significant effect on yield. The extraction of phytochemicals from WCNs has been poorly investigated [53]. Four types of solvents were compared for the recovery of phytochemical from WCN. In this work, methanolic extract of WCNs was found to be a chief source of major phytochemicals. Analogous results were reported by [54]. The variations in extraction yield observed in this study were attributed to the differing polarities of the extraction solvents, as noted in the existing literature. The polarity of the extraction solvent can significantly impact the presence and potency of bioactive components in the extract [53]. These findings are compatible with the yields of extraction from other medicinal plants [54]. WCN green extract showed significantly higher total phenolic acid and total flavonoid content compared to the red variety. TPC and TFC contents reported in the present study were within the range of published values of Indian WCNs [55]. In the present study, lower TPC and TFC contents were consistent with previous data reported by [33]. Both varieties showed the highest TPC and TFC in methanolic extract followed by ethanol > acetone > aqueous. It was previously reported that the highest amount of the total extractable compounds were found in methanolic extracts [56]. These results are in agreement with the earlier reports [57,58] that also found methanol as an efficient solvent. The results of this study clearly show that the phytochemicals in WCN fruit have potent antioxidant activity and that antioxidant activity is positively correlated with total phenolic and flavonoid contents. Phenolic compounds function as reducing agents and hydrogen donors, enabling them to effectively scavenge free radicals. These antioxidants scavenge peroxyl radicals, hydroxyl, hypochlorous acid, and superoxide anions protecting the organism from oxidative damage [59]. To ensure accuracy, two different antioxidant assays, the DPPH free radical scavenging assay and the FRAP radical-scavenging assay, were used to assess the antioxidant activities of the methanolic extract of WCNs. The phenolic extract from WCN green fruit exhibited the highest DPPH radical scavenging activity compared to WCN red fruit. This indicates that locally grown varieties of WCN, especially the green variety, could be a valuable source of natural antioxidants. These results are comparable with results reported in the literature [60]. The highest FRAP value was found in the methanol extract of WCN green fruit as compared to the red fruit, indicating more substantial antioxidant power in the WCN green fruit. These results are in accordance with the literature that genetic and agronomic factors determine the phenolic composition of fruits [61].

## 5. Conclusions

The results highlighted that the proximate, physicochemical, mineral, vitamin, and phytochemical compositions of green and red WCN fruits varied. Both fruits contained bioactive compounds with health benefits, as evidenced by phytochemical screening of their extracts. WCN green fruits exhibited superior antioxidant properties compared to WCN red fruits, with higher levels of DPPH scavenging activity, FRAP value, TPC, TFC, and vitamin C. Moreover, methanol was found to be an effective solvent for extracting terpenoids, alkaloids, flavonoids, and phenolic compounds from WCNs. The extracts showed promising in vitro antioxidant capabilities, suggesting the potential of WCN as a source of antioxidants and anti-inflammatory compounds in the nutraceutical and pharmaceutical industries. These findings demonstrate that WCNs are a significant and promising source of phytochemicals and antioxidant agents.

## Figures and Tables

**Table 1:** Proximate composition of WCN fruits.

WCN Variety	Moisture	Ash	Fiber	Protein	Fat	Starch
	(%)	(%)	(%)	(%)	(%)	(%)
Green	70.23 ± 0.53	1.16 ± 0.07	2.21 ± 0.07	1.72 ± 0.08	0.65 ± 0.01	19.52 ± 0.31
Red	68.74 ± 0.57	1.03 ± 0.03	2.04 ± 0.02	1.18 ± 0.03	0.54 ± 0.05	21.99 ± 0.05
*p*-value	0.020	0.033	0.017	0.001	<0.001	0.009

The experimental data are presented as mean ± standard deviation (SD) of three independent ex-periments. Significant differences between green and red varieties are denoted by *p* < 0.05.

**Table 2:** Physicochemical properties of WCN green and WCN red fruits.

WCN Variety	pH	°Brix	Energy Kcal	Glycemic Index
Green	8.70 ± 0.17	5.20 ± 0.17	112.80 ± 1.55	28.94 ± 0.93
Red	9.63 ± 0.23	6.20 ± 0.26	108.02 ± 1.05	31.34 ± 1.15
*p*-value	0.005	0.005	0.012	0.044

The experimental data are presented as mean ± standard deviation (SD) of three independent experiments. Significant differences between green and red varieties are denoted by *p* < 0.05.

**Table 3:** Mineral contents of WCN green and WCN red fruits.

Minerals	WCN Green	WCN Red
Macro minerals (mg/100 g)		
Na	3.11 ± 0.15 ^a^	2.51 ± 0.11 ^b^
K	392.02 ± 34.13 ^a^	315.01 ± 24.25 ^b^
Ca	49.27 ± 2.01 ^a^	34.20 ± 1.51 ^b^
P	132.04 ± 3.25 ^a^	118.00 ± 2.96 ^b^
Micro minerals (µg/g)		
Fe	43.33 ± 1.12 ^a^	40.10 ± 1.06 ^b^
Mn	79.66 ± 7.15 ^a^	95.00 ± 12.35 ^b^
Cu	23.66 ± 1.76 ^a^	20.33 ± 1.51 ^b^
Zn	31.33 ± 2.50 ^a^	35.33 ± 1.57 ^b^

The experimental data are presented as mean ± standard deviation (SD) of three independent experiments. Mean values in the same row with different superscript indicate significant differences (*p* < 0.05).

**Table 4:** Vitamin contents of WCN green and WCN red fruits.

Verities	WCN Green	WCN Red
Water-soluble vitamin contents		
Ascorbic acid (mg/100 g)	2.10 ± 0.21 ^a^	1.97 ± 0.34 ^b^
Pyridoxine (mg/100 g)	0.29 ± 0.04 ^a^	0.21 ± 0.02 ^b^
Riboflavin (µg/100 g)	ND	ND
Niacin (µg/100 g)	ND	ND
Fat-soluble vitamin contents		
*β*-Carotene (µg/100 g)	46.81 ± 0.18 ^a^	39.31 ± 0.50 ^b^
Retinol (µg/100 g)	ND	ND

The experimental data are presented as mean ± standard deviation (SD) of three independent experiments. Mean values in the same row with different superscript indicate significant differences (*p* < 0.05). ND = Not detected.

**Table 5:** Qualitative phytochemical screening of WCN green fruit with different solvent extracts.

Phytochemical	Observation	Methanol	Ethanol	Acetone	Aqueous
Flavonoids	The color yellow endures	+++	+++	+	−
Alkaloids	An orange solid has formed out of a solution	++	+	+	−
Phenols	A hue of blue is present	+++	+++	+	+
Terpenoids	The color can be described as reddish brown	++	++	−	−
Tannins	The color appears to be a greenish-brown tone	++	+++	+	−
Saponins	An emulsion has been formed	+++	++	++	+
Phlobatannins	The formation of a red precipitate	++	+	+	−
Anthraquinines	The coloration of pink, violet, or red	−	−	−	−

The presence and strength of phytochemical contents can be indicated by the use of a four-point scale, where the symbol “+++” denotes a strong presence, “++” denotes a partially strong presence, “+” denotes a weak presence, and “−” denotes an absence of phytochemical contents.

**Table 6:** Qualitative phytochemical screening of WCN red fruit with different solvent extracts.

Phytochemical	Observation	Methanol	Ethanol	Acetone	Aqueous
Flavonoids	The color yellow endures	+++	+++	++	+
Alkaloids	An orange solid has formed out of a solution	++	+	+	−
Phenols	A hue of blue is present	+++	++	+	+
Terpenoids	The color can be described as reddish brown	+	++	−	−
Tannins	The color appears to be a greenish-brown tone	++	+++	+	−
Saponins	An emulsion has been formed	+++	++	+	+
Phlobatannins	The formation of a red precipitate	++	+	+	−
Anthraquinones	The coloration of pink, violet, or red	−	−	−	−

The presence and strength of phytochemical contents can be indicated by the use of a four-point scale, where the symbol “+++” denotes a strong presence, “++” denotes a partially strong presence, “+” denotes a weak presence, and “−” denotes an absence of phytochemical contents.

**Table 7:** Phytochemical and antioxidant parameters of red and green WCNs.

Parameter	WCN Green	WCN Red
Total phenolics (mg GAE)/g)	91.13 ± 1.06 ^a^	36.60 ± 1.21 ^b^
Total flavonoids (mg QE/g)	69.84 ± 0.08 ^a^	24.19 ± 0.06 ^b^
DPPH assay (% Inhibition)	62.84 ± 0.94 ^a^	50.22 ± 0.79 ^b^
FRAP assay (µM TE/g DE)	42.45 ± 1.45 ^a^	31.72 ± 0.54 ^b^

Each value represents the mean ± standard deviation (SD) of three independent experiments. Mean values in the same row with different superscript indicate significant differences (*p* < 0.05).

## Data Availability

The original contributions presented in the study are included in the article, further inquiries can be directed to the corresponding author.
